# *“There definitely should be some more help for families”:* a call for federal support for families managing pediatric food allergy

**DOI:** 10.1186/s13223-023-00813-3

**Published:** 2023-07-08

**Authors:** Manvir Bhamra, Zoe Harbottle, Michael A Golding, Moshe Ben-Shoshan, Jennifer D Gerdts, Jennifer LP Protudjer

**Affiliations:** 1 Department of Food and Human Nutritional Sciences, University of Manitoba, Winnipeg, MB Canada; 2 The Children’s Hospital Research Institute of Manitoba, Winnipeg, MB Canada; 3 Department of Pediatrics and Child Health, University of Manitoba, Winnipeg, MB Canada; 4 Department of Allergy and Immunology, McGill University, Montreal, QC Canada; 5 Food Allergy Canada, Toronto, ON Canada; 6 George and Fay Yee Centre for Healthcare Innovation, Winnipeg, MB Canada; 7 Institute of Environmental Medicine, Karolinska Institutet, Stockholm, Sweden; 8 501G-715 McDermot Avenue, Winnipeg, MB R3E 3P4 Canada

**Keywords:** Costs, Food allergy, Policy

In the wake of the pandemic, food prices continue to increase in Canada [1], in what has been described as “the largest increase in grocery prices in Canadian history” [2]. While all families have undoubtedly noticed these increasing costs, we posit that families managing food allergy are disproportionately burdened. Indeed, we reported that, in the year prior to the pandemic, compared to families without food allergy, families with food allergy spent an average of $190 more per month on food [3]. In the early months of the pandemic, families managing food allergy reported a further monthly increase in food spending costs of $99-$213, depending on household income [4]. Over the same period, year-over-year wage growth remains below the rate of inflation [5, 6], rendering families in increasingly precarious positions to purchase essential items, such as food.

Since first introduced in 2016, the federally-supported Canada Child Benefit (CCB) has increased, with adjustments made for net family income in the previous tax year [7]. Related programs also exist in most provinces and territories [8]. While designed to help families offset the costs of raising a child to age 18 years, and make strides towards the Government of Canada’s goal of reducing poverty by 50%, by 2030 [7], the funds available to families who face disproportionately higher food costs, as a result of allergist-diagnosed food allergy, which necessitates medical dietary restrictions, have not been taken into consideration when calculating CCB payments. Moreover, unlike other conditions requiring medical dietary restrictions, such as celiac disease [9], no federal tax credits have been made available to families managing food allergy. In the present study, we aimed to describe parental perceptions of needed supports to assist with the costs of food incurred while raising a child with allergist-diagnosed allergy.

## Methods

This study is part of an overarching, mixed methods, intervention study involving Winnipeg families with children age < 6 years, who have allergist-diagnosed cow’s milk allergy. Each family received a home-delivered, subsidy kit of milk allergy-friendly foods (valued at ~$50 per kit) every second week between March and August 2022. Approximately one month prior to the end of the study, a parent from each family completed an in-depth, semi-structured interview (Table 1) moderated by an experienced qualitative interviewer (MG) and supported by a research assistant (MB or ZH). Interviews were audio-recorded and transcribed verbatim. Data were analysed thematically [10] by two research assistants (MB and ZH) who worked independently, and who had regular discussions with two experienced qualitative researchers (MG and JP). Descriptive data, collected at baseline, were analysed using Stata® Version 17.0 (College Station, TX). This study was approved by the University of Manitoba Health Research Ethics Board HS25168 (H2021:340).

**Table 1:** Select questions from the semi-structured interview guide

What would you tell policy makers about this subsidy?
Is a subsidy the “right” way to help families, or are there other approaches that would better suit families? Please explain.

## Results

Eight parents, all from different families, completed qualitative interviews. On average, families were composed of 4.38 ± 1.78 members, and had an average monthly household income of $3764.29. The median index child was age 2 years and with equal numbers of boys and girls represented. All index children had milk allergy; other reported allergies included peanut (4/8), egg (4/8) and soy (each n = 3/8). Parents were, on average, age 29.9 ± 4.4 years, most (n = 5) had post-secondary education and few (n = 2) had food allergy themselves.

In our thematic analysis, we identified one theme: “There definitely should be some more help for families.” This theme, which was indeed a participant quote, captured what parents perceived to be the most meaningful type of support for families with young children managing food allergy. Within this theme, parents described various ways in which they would benefit from financial support to help offset the price of foods, ranging from “*gift cards*” to continued subsidies, such as that provided by our intervention. Interestingly, parents spoke against a tax credit. For example, one parent noted:

*[I] would prefer probably the subsidy like this portion of it rather than a tax credit*.

In contrast, others described how they would have preferred an enhanced ability to select their own foods, rather than being restricted to those provided by the subsidy, as the desire to select their own foods was important. This was captured in quotes from parents who succinctly expressed their sentiments:

*for people, like to pick, whatever they want*

*like the difficulties of finding dairy-free items in Winnipeg*.

While food procurement was perceived as challenging for some families, a more frequently described concern was the cost of allergy-friendly foods, which was cost-prohibitive for some, particularly as the child may refuse the food. As one participant described:

*[Additional support] would be a huge relief for a lot of people especially if allergies are new, like you don’t really want to be spending $7 to $10 on one new product just for your child to say I am not eating it… that is the big stressful part of it*

Another parent also spoke to the high cost of allergy-friendly foods:

*How expensive things really are! There is nothing that has a label of allergy-friendly that is cheap in any way. So trying to, you know, bring in an income and also pay for food at the same time is horrible. So many people go through and I believe [additional support] can help so many people.*

While families in our study appreciated the subsidy, families spoke about what they perceived to be the best way to provide additional support to offset the cost of allergy-friendly foods. These perceptions were elegantly captured in the statement of one participant:

*I would say actually like helping with the cost of food items would be like the best.*

## Discussion and call for action

Herein, we report on the perceived needs, including preferences for additional support, for families whose young children live with allergist-diagnosed cow’s milk allergy. A bi-weekly, home-delivered, subsidy kit of milk allergy-friendly foods was perceived to be beneficial, but not tailored to need or taste preference.

Milk has been described as the most burdensome allergy, owing both to its ubiquity in the food supply chain and the cost of milk-free alternatives [11]. However, we believe that our findings are transferable to other food allergies. While milk is a near-ubiquitous, and often hidden ingredient in many pre-packaged foods [12], we previously reported that families managing pediatric food allergy, regardless of the type of food allergy, are burdened with excess food costs [3, 4]. Based on data in the months prior to the COVID-19 pandemic, families managing food allergy reported excess food costs of $2376.79 Canadian (CAD) annually compared to families who had no medical dietary restrictions (including no food allergy) [3]. To place this financial burden in context, these excess costs correspond to roughly two-thirds (63.1%) of the average monthly household income of participants in the present study. Yet, that excess reflects costs incurred prior to the pandemic. Over the course of the pandemic, food price increases have outpaced salary increase, and indeed are at the highest levels noted in 40 years [13]. Data collected in the early months of the pandemic support that families managing any pediatric food allergy continue to face higher food prices, even beyond those reported in the months prior to the pandemic [4]. While greater increases were reported by higher income families, lower income families nonetheless reported increased costs of, on average, $98.89 CAD monthly [4]. Extrapolated to an annual cost, this pandemic increase totals approximately $1200 CAD, or an additional one-third (31.8%) of the average participant. In brief, families managing pediatric food allergy spend an estimated additional one month’s salary more on food, compared to families who do not manage pediatric food allergy. These estimates are based on data from food allergy of any type, not specific to milk. While milk and its derivatives are a common beverage, food or ingredient in many foods, the collective evidence from our present qualitative analysis, and the above-described cost data provide evidence of need for financial supports for these families.

The participants in our study had an average annual household income of approximately $45,200 CAD, which approximates the low income cut-off for Canadian families of four, in large, urban centres [14], and lower than the average annual household income in Winnipeg (2019: $75,400 CAD [15]). Moreover, study participants lived in Winnipeg, a city which has a lower cost of living overall, and for food, than other large Canadian cites [16], a fact which warrants reflection when considering transferability of our findings to other Canadian centres. Given that the majority (75%) of the children of the participating families were less than age 4 years when the data was collected (2022), these families were not positioned to draw comparisons to their child’s pre-pandemic, i.e. pre-March 2018, food costs.

Based on the qualitative evidence provided herein, taken collectively with findings from our previous work [3, 4, 11], we call on the Government of Canada to commit to the provision of additional funds, delivered through the CCB or similar, to families managing pediatric food allergy, to offset the cost of allergy-friendly food. This governmental commitment would immediately benefit Canadian families whose children have food allergy, but also have downstream sequelae, including a step toward poverty reduction and improved health outcomes.

## Data Availability

Owing to the highly personal nature of qualitative data, requests for data will be carefully vetted by a minimum of three authors.

## Abbreviations

CAD: Canadian dollars
CCB: Canada Child Benefit
